# Neutrophils from Alzheimer’s disease mice fail to phagocytose debris and show altered release of immune modulators with age

**DOI:** 10.3389/fimmu.2025.1672768

**Published:** 2025-10-20

**Authors:** Matthew Kays, Anna Kelly, Farrah McGinnis, Clara Woods, Emily Herron, Candice Brown, Aminata P. Coulibaly

**Affiliations:** ^1^ Department of Neuroscience, Rockefeller Neuroscience Institute, West Virginia University, Morgantown, WV, United States; ^2^ Morrisey School of Arts and Science, Boston College, Chestnut Hill, MA, United States; ^3^ Departments of Anesthesia and Neurology, Indiana University School of Medicine, Stark Neurosciences Research Institute, Indianapolis, IN, United States

**Keywords:** age effect, Alzheimer’s disease, AD neutrophils, phagocytosis, amyloid beta, cytokine production, 3xTg mouse

## Abstract

Recent reports show that neutrophil activity plays a role in the cognitive decline associated with Alzheimer’s Disease (AD). There is evidence of altered functions in neutrophils isolated from AD patients. Whether these altered functions are inherent to the AD disease state is unknown. The goal of this study was to determine if neutrophil functions are altered in AD mice and if these changes occur only after symptoms appear. To address this hypothesis, we used a primary neuronal culture model, generated from 3xTg perinatal mice, since AD is considered a CNS disease. The 3xTg primary neuronal culture gradually increase the release of Aβ (40 and 42) as the culture ages. To assess neutrophil functions, neutrophils isolated from young male/female mice (3–6 months of age) or aged male/female mice (16–18 months of age) of WT or 3xTg mice were exposed to 3xTg primary neuronal cultures. To assess phagocytosis, we characterized the effect of neutrophils on pathogenic amyloid beta (Aβ) 42 levels. To assess the levels of immune modulators (cytokines, chemokines, growth factors, NETosis, and neutrophil granular content), culture media were assessed using Luminex multiplex assay. Our results show that neutrophils from young AD mice have impaired phagocytosis, as observed in a decreased ability to remove Aβ and cellular debris *in vitro*. Neutrophils from young AD mice also increased release of pro-inflammatory cytokines, granule content, and NETs in 3xTg primary neuronal cultures. Interestingly, neutrophils from aged 3xTg mice decreased Aβ levels in culture and expression of proinflammatory cytokines when compared to neutrophils from aged WT mice. These neutrophils increased their release of granule content and NETs in 3xTg primary neuronal cultures. These data show that in AD neutrophil function is altered in (young mice) and diseased (old mice) stage.

## Introduction

With the increasing incidence of Alzheimer’s Disease (AD), there is dire need to fully understand its etiology with the hope of finding effective treatments. It is understood that AD is a progressive disease with onset long before cognitive symptoms manifest (prodromal stage). AD symptomology is characterized by abnormal cognitive and behavioral changes. These changes, including memory loss, diminishes a person’s quality of life and contribution to society. AD is characterized, pathologically, by the accumulation of two key proteins, extracellular amyloid beta (Aβ) and intracellular hyperphosphorylated Tau (p-Tau) (1). These two proteins have been shown to be highly toxic (2). There is mounting evidence of peripheral immune recruitment to the brain in AD (3). Neutrophils, a peripheral immune cell type, are found around Aβ deposits in the brain of AD patients (4). Several recent studies have demonstrated neutrophil recruitment to the brain vasculature in several rodent AD models (5–7). Interestingly, two studies have shown that neutrophils isolated from AD patients show impaired phagocytosis and increased release of inflammatory molecules (8, 9). These data suggest that in AD, neutrophil functions are altered. Whether these cellular changes are present in pre-clinical AD models or occur in the prodromal phase of the disease are currently unknown. In this study, we hypothesized that neutrophil from Alzheimer’s Disease pre-clinical models show impaired function and activity in the prodromal and disease stages.

To test this hypothesis, we used the transgenic 3xTg mouse model of AD. This model contains 3 mutations associated with familial forms of AD. These mutations are the Swedish mutation in the amyloid precursor protein (K670N/M671L), a mutation in the presenilin protein (M146V), and a mutation in the Tau protein (P301L). The 3xTg mouse is slow progressing with Aβ detection, cognitive decline, and neuroinflammation at 6 months of age (10). Using cell culture, histology, cytokine assays, and amyloid ELISA, our data showed that neutrophils from 3xTg mice failed to phagocytose Aβ and cellular debris, showed altered cytokine production, and differed from WT derived neutrophils in both young (prodromal) and aged (diseased) mice.

## Material and methods

### Animals

Young (3–6 months) and aged (16–18 months), male and female 3xTg (B6;129-Tg(APPSwe,tauP301L)1Lfa *Psen1^tm1Mpm^*/Mmjax), C57Bl/6 wildtype, and CVN (APPSwDI-Nos2^-/-^) mice were used for this project. 3xTg and CVN perinatal day 0 were used for primary neuron culture generation. Mice were kept on a 12 hour:12-hour light cycle at room temperature (22-25°C). Food and water were provided *ad libitum*. All experiments were done with the approval of West Virginia University Animal Care and Use Committee.

### Primary neuron cultures

Brains of 3xTg and CVN P0 pups were collected to generate primary cortical neuronal cultures as previously described (11). P0 pups were anesthetized using hypothermia (10 min in ice) and heads removed. Brains were removed from the pups, and brainstem and midbrain structures were discarded. Remaining brain samples were digested in a medium containing DMEM/F12, glucose, Glutamax, Na+/pyruvate, FBS and penicillin/streptomycin. Brain samples were centrifuged and resuspended in the same media. 12 well plates, coated with poly-D-Lysine, were seeded at a density of 2x10^5^ cells/well. After a 2-hour incubation, media was removed and replaced with media containing Neurobasal, Glutamax, B27, and penicillin/streptomycin. Cultures were maintained for 4 weeks with one third media replacement every 3–4 days. Starting at week 2, 500μL of media was collected from each well weekly and frozen for Aβ ELISA and Luminex assay. Of note, we did not assess the sex of the pups used for cultures. However, across 26 litters from 3xTg breeders 48.1% were male and 51.9% were female. We therefore postulate that the pups used for our experiments were half male and half female.

### Neutrophil isolation

For all genotypes, 2 male and 2 female mice (young or aged) were used for neutrophil isolation. The animals were euthanized using 5% isoflurane in a drop container for 3–5 min followed by cervical dislocation. Tibial and femoral bones were dissected, and bone marrow collected. Neutrophils were isolated using the MojoSort Neutrophil Isolation Kit (BioLegend, 480058), according to the manufacturer’s protocol. The MojoSort kit enriches neutrophils to 80% in our bone marrow isolate with the remaining 20% consisting of mostly blasts and few monocytes, lymphocytes and Ly6G- granulocytes (**Supplementary Figure 1A**). Isolated neutrophils were added to the primary neuron culture at the end of week 4 in duplicate. Neutrophils were seeded at a density of 2x10^5^ cells/well. Co-cultures were incubated for 6 hours as neutrophils have a short life span *in vitro* and we wanted to avoid neutrophil death as a confounding factor (12). Media was collected from each well after the incubation period, and plates were fixed with 4%PFA for IHC staining.

### Flow cytometry

Bone marrow was collected by flushing the tibia and femur with RPMI+EDTA using a 1mL insulin syringe. For blood collection, 100µL of blood was collected from the left ventricle with a 0.3mL insulin syringe containing RPMI+EDTA solution, followed by RBC lysis using ACK lysis buffer. Prior to staining, cell number was assessed in all samples using a ThermoFisher Countess II Automated Cell Counter. Samples were treated with a 1:100 dilution of Fc block (BioLegend, 156603) then stained for CD45 (BioLegend, 157211), Ly6G (BioLegend, 127607), and Live/Dead (ThermoFisher, L34960). Stained samples were analyzed using a LSRFortessa. Cell gating and quantification were performed using the FlowJo software. The gating strategy consisted of selecting the live cell population (Live/dead negative), singlets, and CD45+. Of the CD45+ cells, Ly6G+ cells were selected to determine the number of neutrophils present. Ly6G- cell population was gated using SSC and CD45 to characterize the remaining cell populations (lymphocytes, blasts, monocytes, and non-neutrophil granulocytes).

### ELISA assays

ELISA plates for Aβ40 (Abcam, ab193692) and Aβ42 (ThermoFisher, KHB3441) were used to assess soluble Aβ levels in supernatant collected from each pup culture throughout the four weeks. The manufacturer protocols for the Aβ42 ELISA and Aβ40 ELISA kits were followed. Briefly, antibody solutions were added to all wells (standards, controls, and samples) and incubated for 3 hours. The wells were washed, HRP-Streptavidin solution added, and incubated for 45 minutes. The plates were then washed, and stabilization chromogen added. Plates were read using a plate reader (SpectraMax iD3) at 450nm absorbance.

### Luminex

15-plex Luminex assays (bio-techne, CUSTOM-LXSA-M15) were used to measure secretions of immune modulators in supernatants collected from co-cultures experiments. The proteins assessed were pro-inflammatory cytokines (IL-1beta, IL-6, IL-17, TNFalpha, IL-alpha), anti-inflammatory cytokines (IL-4, IL-10), chemokines (CCL2, CXCL1, CXCL12), growth factors (G-CSF, IGF-1, beta-NGF), a marker for degranulation (MMP-9), and a marker for NETosis (S100A8). Luminex assays were run according to manufacturer’s protocol by the Flow Cytometry and Single Cell core facility at West Virginia University Health Science Center. Assays were run on the Luminex MAG PiX instrument using the Xponent software version 4.3.

### Free DNA detection

The concentration of free DNA in supernatants from cultures was determined using the Quant-iT PicoGreen dsDNA assay kit (ThermoFisher, P11496). Assay was performed according to manufacturer’s protocol. Plates were read using a plate reader (SpectraMax iD3) set to fluorescence at 480 excitation and 520 emission.

### Immunohistochemistry staining

Paraformaldehyde fixed culture plates were stained using basic immunofluorescence staining protocols. Briefly, cultures were incubated in blocking buffer containing 5% normal goat serum and 0.1% Triton X in 1xPBS for 30 minutes; followed by incubations with primary antibody cocktails (Aβ37-42 (Cell Signaling, 8243), neutrophils (Ly6B; ThermoFisher, MA5-16541), neurons (beta III Tubulin; Abcam, ab78078), and astrocytes (GFAP; ThermoFisher, PA1-10004)) for two hours. Cultures were subsequently incubated with the appropriate secondary antibody cocktail (ThermoFisher) in 1xPBS for one hour. Cultures were imaged on the Echo microscope, converted to multi-channel images using ImageJ, and quantified using IMARIS.

### Quantification using IMARIS

Images were analyzed and quantified using IMARIS. 3D reconstructions were made using the surface analysis tool on IMARIS. 3D reconstructions were used to determine the number of neutrophils (Ly6B+) and the size (fragment area, µm^2^) of Aβ plaques (example of 3D reconstruction in **Supplementary Figure 1F**).

### Aβ plaque and debris analysis

After surface generation, IMARIS generated an excel file with all the detected plaques/debris and their corresponding volume in voxels. Using the frequency distribution analysis tool on GraphPad Prism, with bin width set to 50 voxels, the number of plaques/debris with sizes between 0–600 voxels was determined for each bin. Culture duplicates were averaged to generate one set of frequency values. Frequency values (“number of values”) were presented as histograms. Internalized and non-internalized Aβ fragments were determined by the distance between the neutrophil surface and the Aβ surface. Surfaces with distances more than 1µm away from neutrophil (Ly6B+) edge were considered outside of neutrophils and amyloid beta surfaces below 0 µm (0 indicated the edge of the neutrophil surface) were labeled inside neutrophils (**Supplementary Figure 1F**).

### Statistical analysis

All statistical analyses were performed using GraphPad Prism. For all data generated, no-neutrophil controls were analyzed for statistical difference in the young and aged groups. If no significance was observed (as with the no neutrophil control for the young male and female Aβ experiments) controls were combined to generate one set of controls. If they were statistically significant (as with the aged group), controls were kept separate for analysis. A two-way ANOVA was used for comparisons between the control and neutrophil groups for the size distribution of internalized Aβ. One-way ANOVA was used for comparisons between the control and neutrophil groups for ELISA data. A one-way ANOVA was performed on raw concentration values for each analyte from the Luminex Assay. A *post-hoc* analysis (specified in each figure legend) was performed for each ANOVA doing pairwise comparison between each group. Pairwise significance is only noted when ANOVA is significant. Significance was ascribed at p<0.05.

## Results

### Primary neuron cultures from 3xTg mice release increasing levels of Aβ *in vitro*

**Figure 1:**
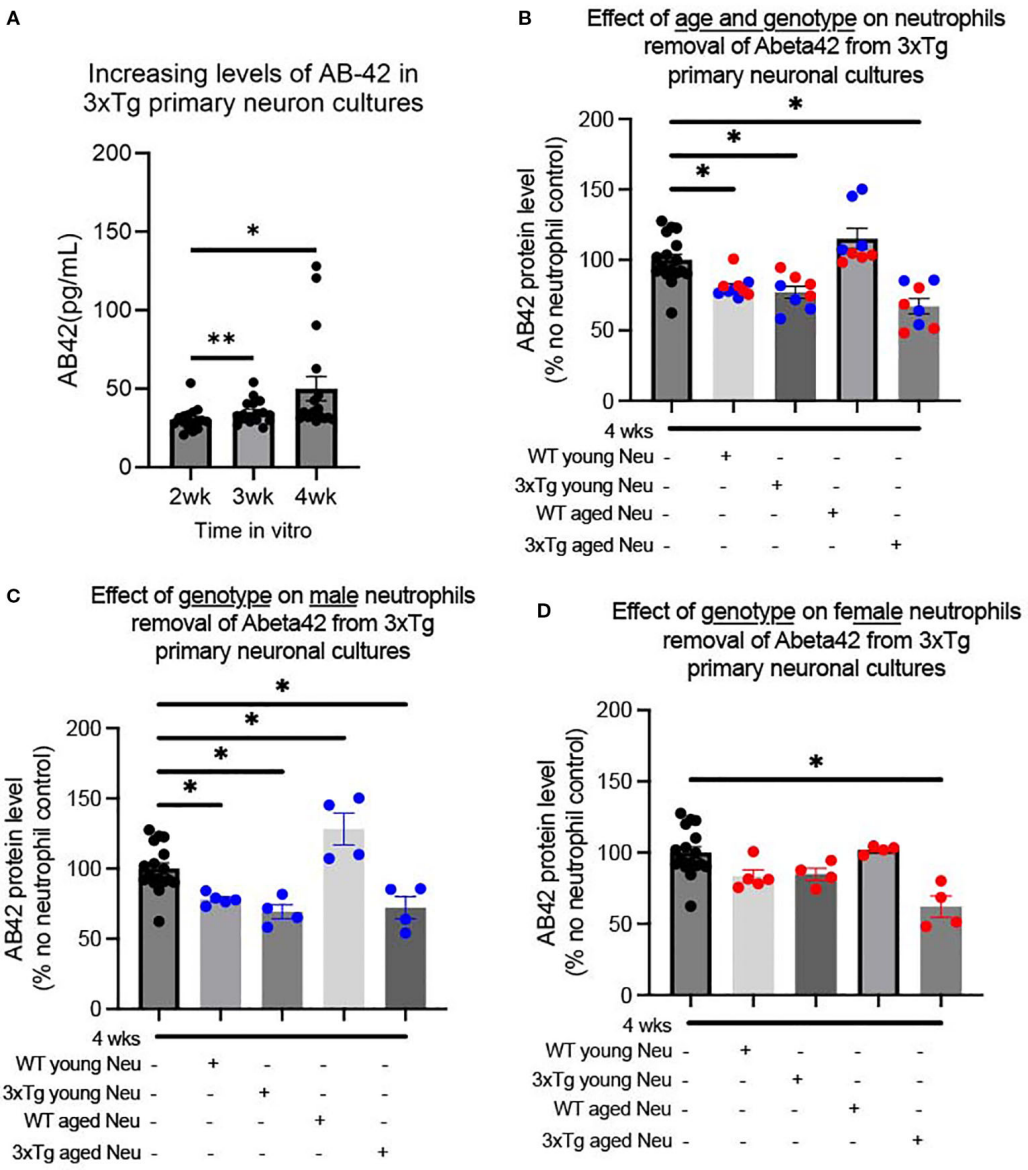
Both genotype and sex affect neutrophil removal of soluble Aβ-42 from 3xTg primary neuronal cultures. Primary neuronal cultures, generated from P0 3xTg mice, produced increasing levels of Aβ-42 over 4 weeks in vitro **(A)**. The addition of bone marrow neutrophils from young and aged mice significantly decreased Aβ-42 levels in 4week cultures, except those derived from WT aged mice **(B)**. Male neutrophils from WT/3xTg young and 3xTg aged neutrophils decreased the level of Aβ-42, while WT aged neutrophils increased its levels **(C)**. For female mice, only neutrophils from aged 3xTg mice significantly decreased the levels of Aβ in cultures **(D)**. Dots represent the number of distinct pup cultures exposed to neutrophils isolated from either male or female, WT or 3xTg mice. Of note, each sex experiments were conducted in duplicate and averaged. One-way ANOVA was conducted followed by Dunnett’s multiple comparison test for post-hoc analysis. * p<0.05, ** p<0.005. In bar graphs, red dots are cultures exposed to female-derived cells and blue dots are cultures exposed to male-derived cells.

Previous studies showed very little astrocyte contamination of primary neuronal cultures generated from P0 pups (13), with cortical cultures having 4% and hippocampal cultures 28% astrocytes (GFAP) contamination (13). Therefore, we characterized the GFAP content observed in our primary 3xTg neuronal cultures (**Supplementary Figure 1B**). In cultures generated from 3xTg pups, GFAP+ cells made up on average 15% of cells in cultures (**Supplementary Figure 1C**). To determine if 3xTg primary neuronal cultures release Aβ *in-vitro*, culture media was collected at 2, 3, and 4 weeks *in vitro*. Our data showed that as early as 2 weeks *in vitro*, primary 3xTg neuronal cultures release detectable levels of Aβ (**Figure 1A**). Characterization of two Aβ species, show a gradual increase of soluble Aβ-42 (one-way ANOVA: p=0.013, F(1.06, 17.01)=7.31) and steady levels of soluble Aβ-40 (**Supplementary Figure 2A**). Aβ-42 levels were significantly increased at both 3wks (p=0.01) and 4wks (p=0.02) from the 2wks time point *in-vitro* (**Figure 1A**).

### Neutrophils from female 3xTg mice fail to reduce soluble Aβ42 levels in culture

Of note, the bone marrow of 3xTg mice contain significantly less neutrophils than WT bone marrow (**Supplementary Figure 1D**). Specifically, the number of neutrophils in young 3xTg bone marrow was similar to the number observed in aged WT mice (**Supplementary Figure 1D**). This decreased bone marrow is a result of increased neutrophil release in the young 3xTg mouse, as indicated by increased neutrophils in the blood of the young 3xTg mouse (**Supplementary Figure 1E**). This suggests an increased inflammatory state in 3xTg mice at a young age (14, 15). These data suggest there are inherent physiological changes in 3xTg mice that precede the start of disease symptomology.

We determined whether the presence of neutrophils would affect the levels of Aβ in the 3xTg primary neuronal cultures. At 4 wks, primary neuron cultures were exposed to neutrophils (1:11 ratio) from WT and 3xTg mice. The addition of neutrophils significantly decreased soluble Aβ-42 levels (One-way ANOVA: p <0.0001; F(4,46)= 14.29; **Figure 1B**) with no change to soluble Aβ40 levels (**Supplementary Figure 2B**). Further characterization showed that neutrophils from young WT (p=0.010), young 3xTg (p=0.003), and aged 3xTg (p=0.00002) significantly decrease soluble Aβ-42 levels, while those from WT aged mice did not (**Figure 1B**). With aged-derived neutrophils, cultures with neutrophils from 3xTg mice had significantly less soluble Aβ-42 compared to those from WT (p<0.05).

To determine if sex or age influenced the effect of neutrophils on Aβ levels, cultures were incubated with neutrophils isolated from male or female mice from WT/3xTg, young/aged mice. For male derived neutrophils, cells from WT young (p= 0.039), 3xTg young (p= 0.005), and 3xTg aged (p=0.013) significantly decreased Aβ-42 levels in culture (**Figure 1C**). Interestingly, neutrophils from WT aged male mice increased the levels of Aβ-42 levels *in-vitro* (p= 0.011) and those from 3xTg young male mice increased Aβ-40 levels (**Supplementary Figure 2C**). On the other hand, only neutrophils from 3xTg aged female mice significantly decreased Aβ-42 levels (p=0.0001; **Figure 1D**) with no effect on Aβ-40 levels (**Supplementary Figure 2D**). These data suggest that the activity of neutrophils changes based on the disease state and sex of the mouse.

### Neutrophils from young 3xTg mice fail to phagocytose Aβ plaques and debris in culture

**Figure 2:**
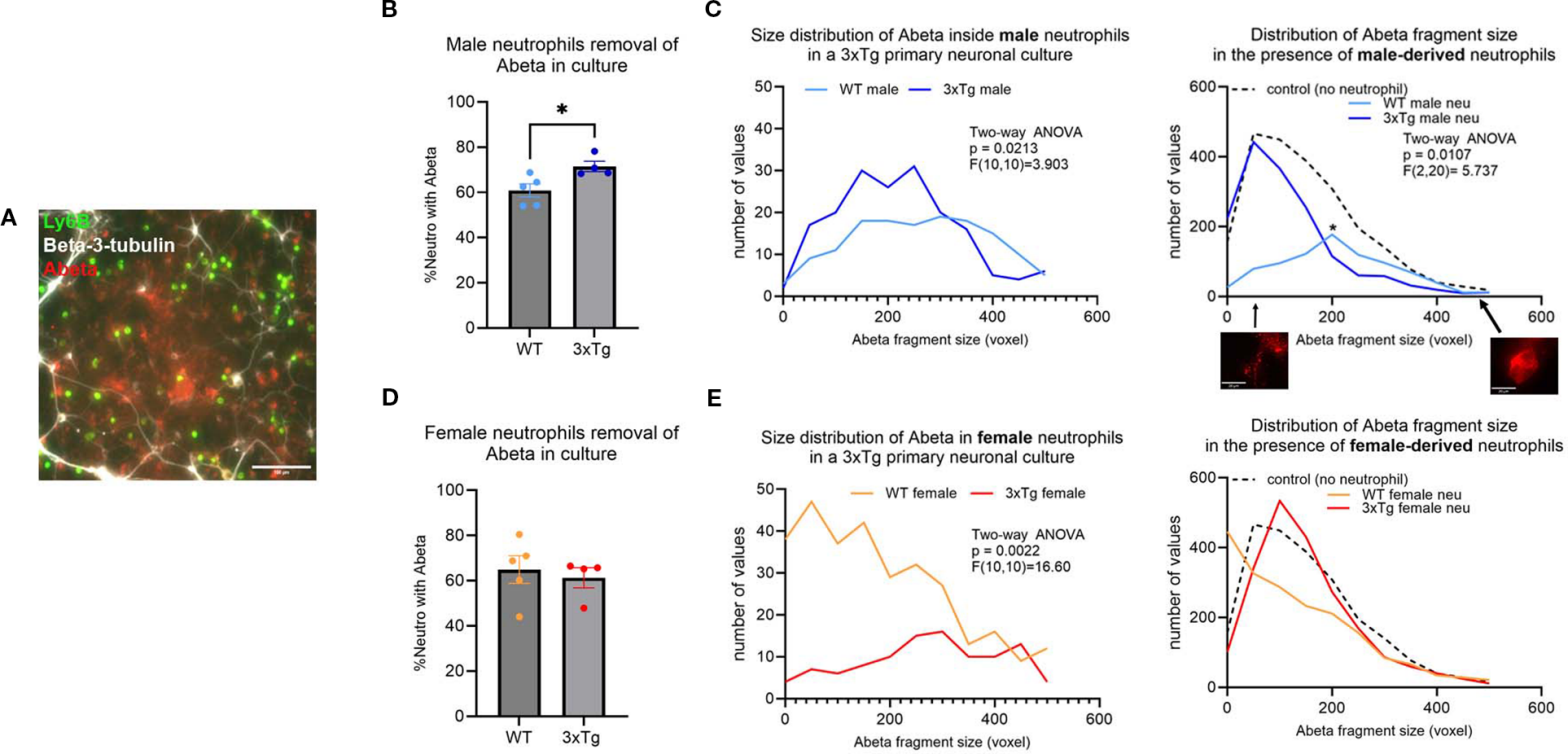
Neutrophils derived from young (3-6 months) WT mice are better at removing Aβ plaques in vitro. A representative micrograph depicting the presence of Aβ plaques (red) in 3xTg primary neuronal cultures (white; beta-3-tubulin) after the addition of isolated neutrophils (green; Ly6B) **(A)**. More neutrophils from young 3xTg male mice contained Aβ than those from young WT male mice **(B)**. Further analysis of the size of the Aβ fragments inside the cells show more 3xTg neutrophils contain Aβ fragments **(C)**. Overall, only young WT male neutrophils significantly decreased the number and Aβ plaques in vitro **(C)**. The number of neutrophils from young WT and 3xTg female mice with internalized Aβ was not different **(D)**. WT female neutrophils internalize more Aβ fragments than those from the 3xTg with no effect on the distribution and number of Aβ plaques in culture **(E)**. Scale bars: A= 100 µm; C=20 µm. Each dot represents data from a pup culture (n=4) exposed to neutrophils isolated from either male or female, WT or 3xTg mice. Data represented in C and E are from cultures used for B and D, respectively. ANOVA performed followed by Tukey’s multiple comparison test. *, p<0.05.

To assess the activity of neutrophils in the 3xTg primary neuronal cultures, we characterized phagocytosis by determining the percentage of neutrophils with internalized Aβ fragments (**Supplementary Figure 1F**), the size distribution of internalized fragments, and the number of Aβ plaques (**Figure 2A**). 80% of Aβ plaques were found outside of beta-3-tubulin+ neurons (**Supplementary Figure 1G**). On average 60% of neutrophils from young mice contain Aβ fragments (**Figures 2B, D**), which was significantly increased to 75% in neutrophils from young 3xTg male mice (**Figure 2B**; p=0.0287). Neutrophils from young male 3xTg mice contained fewer Aβ fragments than those from WT male mice (**Figure 2C**; p=0.0213). To determine if this internalization led to a decrease in plaque burden, we assessed the distribution and range of remaining Aβ plaques in culture. Neutrophils from young WT mice had fewer Aβ fragments internalized but showed the largest reduction in Aβ plaque number. This suggests a continuous internalization and breakdown of Aβ by these cells. On the other hand, neutrophils from young male 3xTg mice failed to decrease the number of Aβ plaques in cultures, even with an increased number of internalized fragments. This suggest that these cells can internalize Aβ but may fail to degrade it, leading to a cytoplasmic accumulation over time. Interestingly, the quantity of small (<200 voxels) and large (>200) plaques were decreased by neutrophils from young WT male mice, while neutrophils from young 3xTg male mice only decreased the number of large plaques (**Supplementary Figure 3A**). Neutrophils from young female 3xTg mice had fewer internalized Aβ fragments compared to those from young WT female mice (**Figure 2E**; p=0.0022). Interestingly, neither decreased the distribution or number of Aβ plaques in culture. Neutrophils from young female WT and 3xTg mice had no effect on the number of small or large plaques in culture (**Supplementary Figure 3B**). These data suggest that sex contributes to neutrophil modulation of Aβ levels *in vitro*.

To determine if the deficit in phagocytosis is specific to Aβ, we assessed neutrophil removal of cellular debris. For this experiment, we generated primary neuronal cultures from the CVN-AD (16) mouse, a vascular model of AD (17). Primary neuronal cultures from this strain died at 3 weeks in culture, leading to a plate with neuronal debris and fragments (**Supplementary Figure 4A**). Like neutrophils from 3xTg young male mice, neutrophils isolated from the young male CVN mice fail to clear cellular debris (**Supplementary Figure 4B**). Neutrophils from young WT male mice removed a significant number of cellular debris in comparison (**Supplementary Figure 4B**; p<0.001). Neutrophils from young WT female mice removed cellular debris efficiently, while those from the young female CVN mice failed to do so (**Supplementary Figure 4B**; p<0.0001). These data further support a phagocytic deficit in neutrophils from AD mouse models.

### Neutrophils from aged mice fail to clear Aβ in a 3xTg primary neuronal culture

In young mice, ~60-70% of neutrophils contained internalized Aβ fragments. This was significantly decreased in aged mice, with only ~40-50% containing Aβ fragments (**Supplementary Figure 1H**; Two-way ANOVA: p=0.0019, F (1,30) =11.63)). This suggests that age influences neutrophil phagocytosis regardless of disease state.

**Figure 3:**
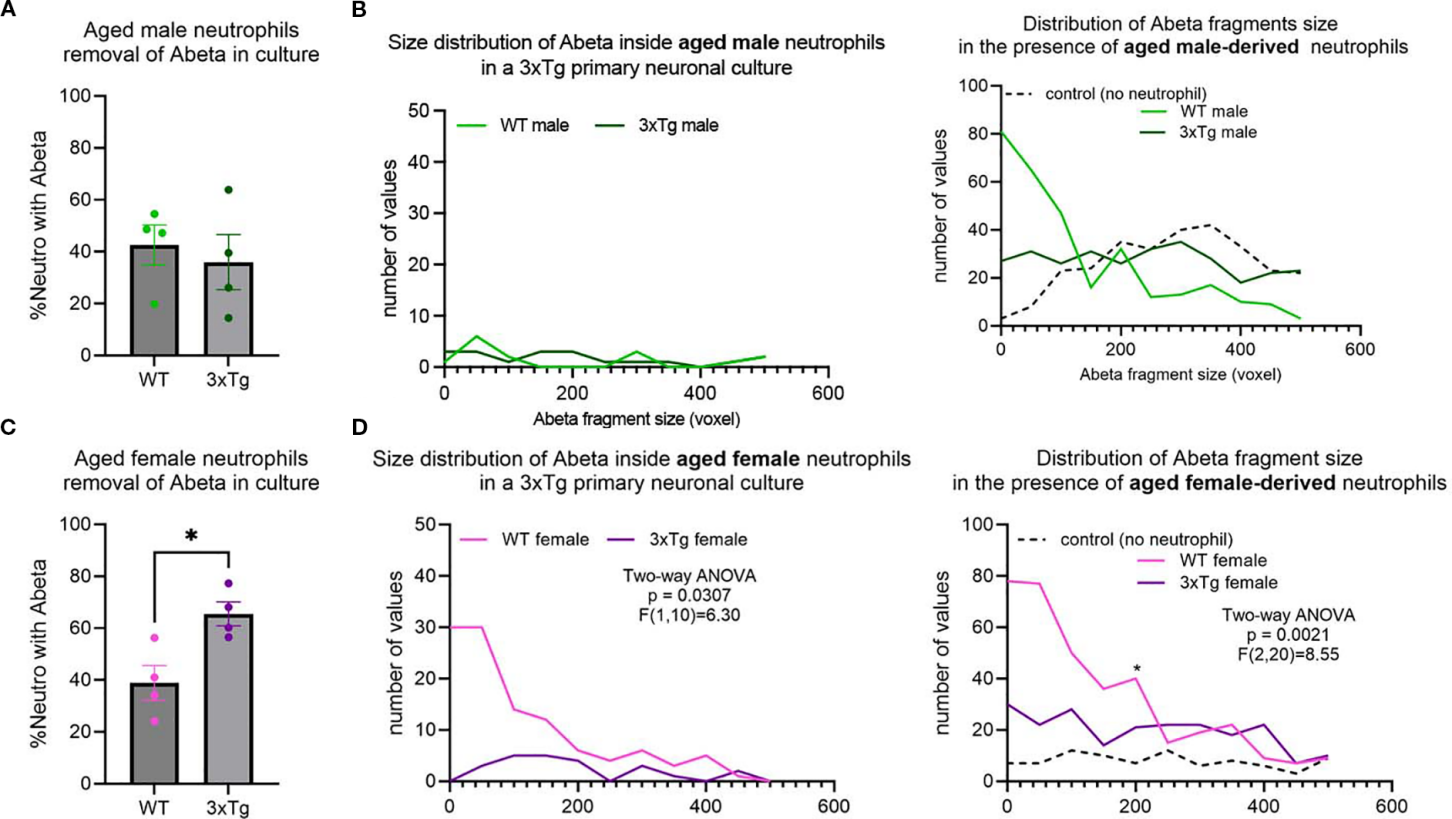
Age alters neutrophils ability to remove Aβ in vitro. No difference in neutrophils internalizing Aβ from WT or 3xTg aged mice **(A)**, with no change in the distribution and number of Aβ fragments inside aged neutrophils or their effect on overall Aβ plaques in cultures **(B)**. More neutrophils from aged 3xTg female mice contained Aβ fragments compared to those from the aged WT female **(C)**. Further analysis showed more Aβ fragments inside neutrophils from aged 3xTg female **(D)**. Interestingly, the presence of neutrophils from aged WT female mice increased the number of Aβ plaques in cultures **(D)**. Each dot represents data from a pup culture (n=4) exposed to neutrophils isolated from either male or female, WT or 3xTg mice. Data represented in B and D are from cultures used for A and C, respectively. *, p<0.05.

Characterization of neutrophils from aged mice showed an abrogation of the changes observed in the cells derived from young mice (**Figure 2**). Specifically, the 3xTg specific changes observed in the male mice were gone (**Figures 3A, B**). On the other hand, neutrophils from 3xTg aged female mice showed a significant increase in the number of cells internalizing Aβ compared to neutrophils from aged WT mice (**Figure 3C**; p=0.0172). Interestingly, neutrophils from WT aged female mice led to an increase in Aβ plaques deposit (**Figure 3D**; compared to no neutrophil control p=0.0010). When comparing small and large plaques, the presence of aged WT male neutrophils increased the number of small plaques and decreased the number of large plaques (**Supplementary Figure 3C**). The presence of neutrophils from aged 3xTg male mice led to a small increase on the number of small plaques, with a small decrease in the number of large plaques (**Supplementary Figure 3C**), suggesting a dampening of the effect from neutrophils from aged WT mice. Neutrophils from aged female WT and 3xTg mice led to increases in the number of both small and large plaques in culture (**Supplementary Figure 3D**). These data suggest that age directly affect how neutrophils influence the build-up of Aβ plaques.

### Both age and animal disease state alter neutrophils release of immune modulators

Using a 15-plex immune array we characterized the production of pro-inflammatory cytokines, anti-inflammatory cytokines, growth factors, chemokines, and markers of degranulation and NETosis in 3xTg cultures exposed to neutrophils isolated from WT/3xTg, young/aged mice.

**Figure 4:**
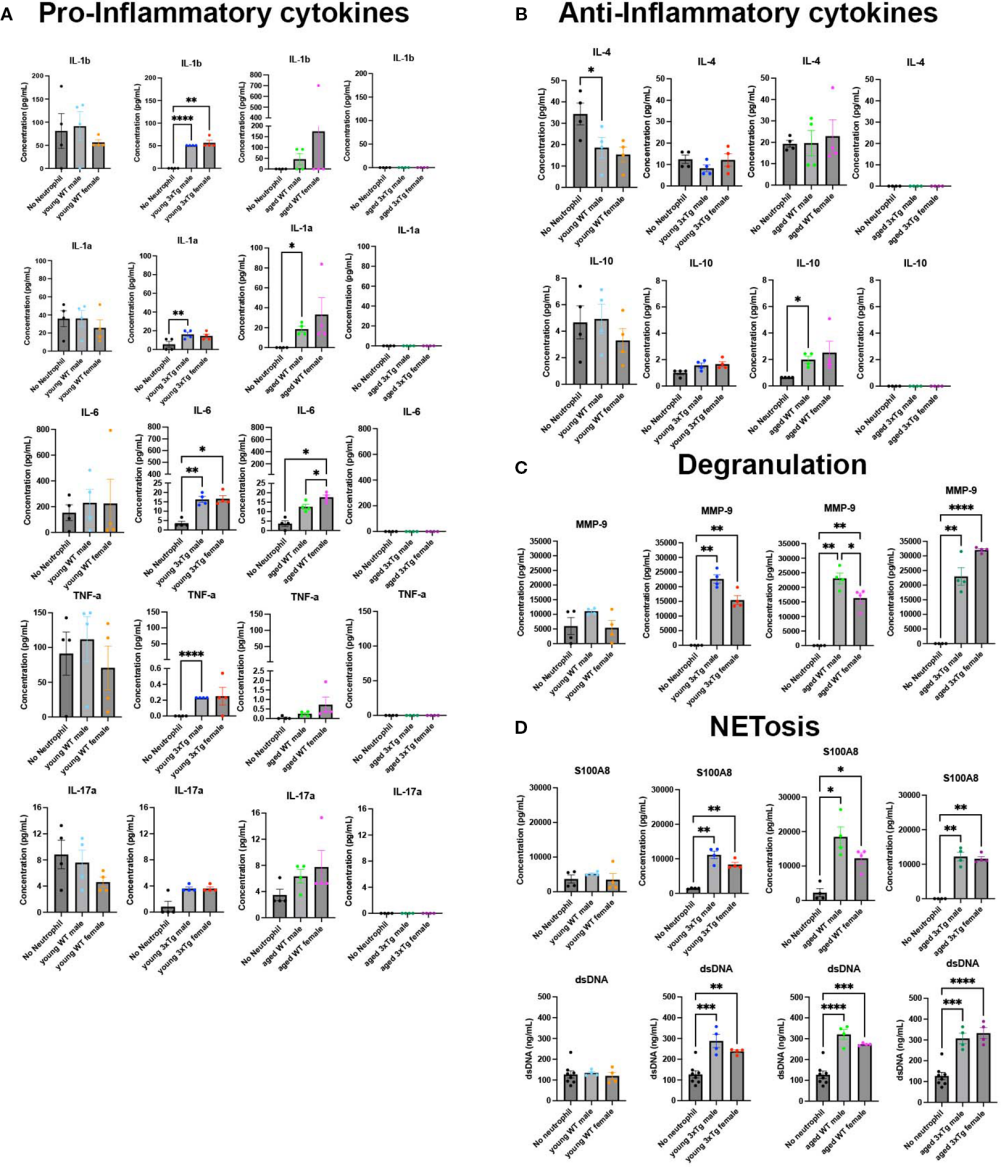
Neutrophils from 3xTg mice have altered release of immune modulators in 3xTg primary neuronal cultures. Culture media contained higher levels of pro-inflammatory cytokines with neutrophils isolated from young 3xTg mice and aged WT mice **(A)**. This increase is absent in cultures with neutrophils from young WT mice and aged 3xtg mice **(A)**. IL-4 is decreased when young WT male neutrophils are added to cultures while IL-10 is increased in the presence of neutrophils from aged WT male mice **(B)**. Markers of degranulation **(C)** and NETosis **(D)** were increased in cultures with all neutrophils except those from young WT mice. Each dot represents data from a pup culture (n=4) exposed to neutrophils isolated from either male or female, WT or 3xTg mice. Neutrophil conditions were compared to their corresponding no neutrophil conditions. One-Way ANOVA was performed followed by Tukey’s multiple comparison test was for post-hoc analysis. *, p<0.05.

It is evident that 3xTg cultures exposed to neutrophils isolated from young 3xTg mice (male and female) have significantly higher levels of pro-inflammatory cytokines (IL1-b, IL1-a, IL6, TNF-a, IL17a) (**Figure 4A**), and no change in the levels of anti-inflammatory cytokines (**Figure 4B**). These cultures also showed increased presence of MMP9, a neutrophil granule protein (**Figure 4C**), and increased NET production, as denoted by increased levels of S100A8 and free DNA (**Figure 4D**). Cultures exposed to neutrophils from young WT mice had no effect on cytokines, MMP9, or NETs levels in the culture media. These data suggest that neutrophils isolated from 3xTg young mice generates an inflammatory environment in the presence of 3xTg neurons.

On the other hand, exposure of 3xTg neuronal cultures to aged WT neutrophils led to an increase in the pro-inflammatory cytokines IL-6 and IL1-a, and the anti-inflammatory cytokine IL-10 (**Figures 4A, B**). Interestingly, cultures with neutrophils from aged 3xTg mice showed no effect on cytokine levels, a direct opposite of what is observed in cultures with neutrophils from young 3xTg mice. Markers of degranulation, MMP-9, and NETosis, S100A8 and free DNA, were significantly increased in 3xTg cultures exposed to neutrophils from both aged WT and 3xTg mice (**Figures 4C, D**). Since increased free DNA correlates with increased S100A8, a neutrophil intracellular protein, it is likely the increase in free DNA is due to neutrophil death. It is however possible that neuronal death could contribute to free DNA in culture. Our analysis found no change in neuron density in cultures when neutrophils are added (**Supplementary Figure 5A**). Therefore, we postulate that free DNA from neuronal death was minimal. These data suggest that the 3xTg disease state significantly alters the activity and response of neutrophils with age.

Chemokines and growth factors were also detected in culture media. The levels of G-CSF increased in 3xTg cultures with the addition of young 3xTg male neutrophils (**Supplementary Figure 5B**). Young 3xTg male and female neutrophils both decreased the levels of CXCL1 while aged 3xTg female neutrophils decreased the level of CXCL12 in culture (**Supplementary Figure 5C**).

## Discussion

The goals of the present study were to determine if neutrophils from AD mice were functionally different than those from WT mice. This was done by investigating the ability of neutrophils from AD mice to clear neuron derived Aβ and determine how age influences this function. Our data showed a clear sex difference in young neutrophils ability to interact with the pathogenic form of Aβ. Neutrophils from young male mice, regardless of disease state, remove more Aβ than neutrophils from young female mice. This sex difference is lost with age. In the aged-derived cells, only neutrophils from the 3xTg mice, male and female, significantly decrease Aβ levels. All 3xTg neutrophils showed impaired phagocytosis regardless of age. Our data also show that neutrophils from young 3xTg mice release more pro-inflammatory cytokines, granule proteins, and NETs in 3xTg neuronal cultures than WT neutrophils. Neutrophils from aged 3xTg mice had increased release of NETs with no release of cytokines in 3xTg neuronal cultures, a result opposite to those from aged WT mice. These data clearly demonstrate that neutrophils from both young and aged AD mice have altered function from WT mice.

Neutrophils have been implicated in AD pathology. Neutrophils infiltrate the brain in various AD mouse models including APP/PS1, 3xTg, and 5xFAD (4, 18, 19). This infiltration was linked to increased microglia activity via release of CAP37 in 3xTg mice at 12 months of age (4). Our data show that neutrophils from aged 3xTg mice have increased release of MMP9 and NETs, which can increase microglia activity and inflammation (20). Neutrophil proteins co-localized with Aβ plaques (18). Neutrophils migrated towards Aβ plaques after infiltrating the AD brain (18). Our study showed that neutrophils not only interact but also phagocytose Aβ fragments.

**Figure 5:**
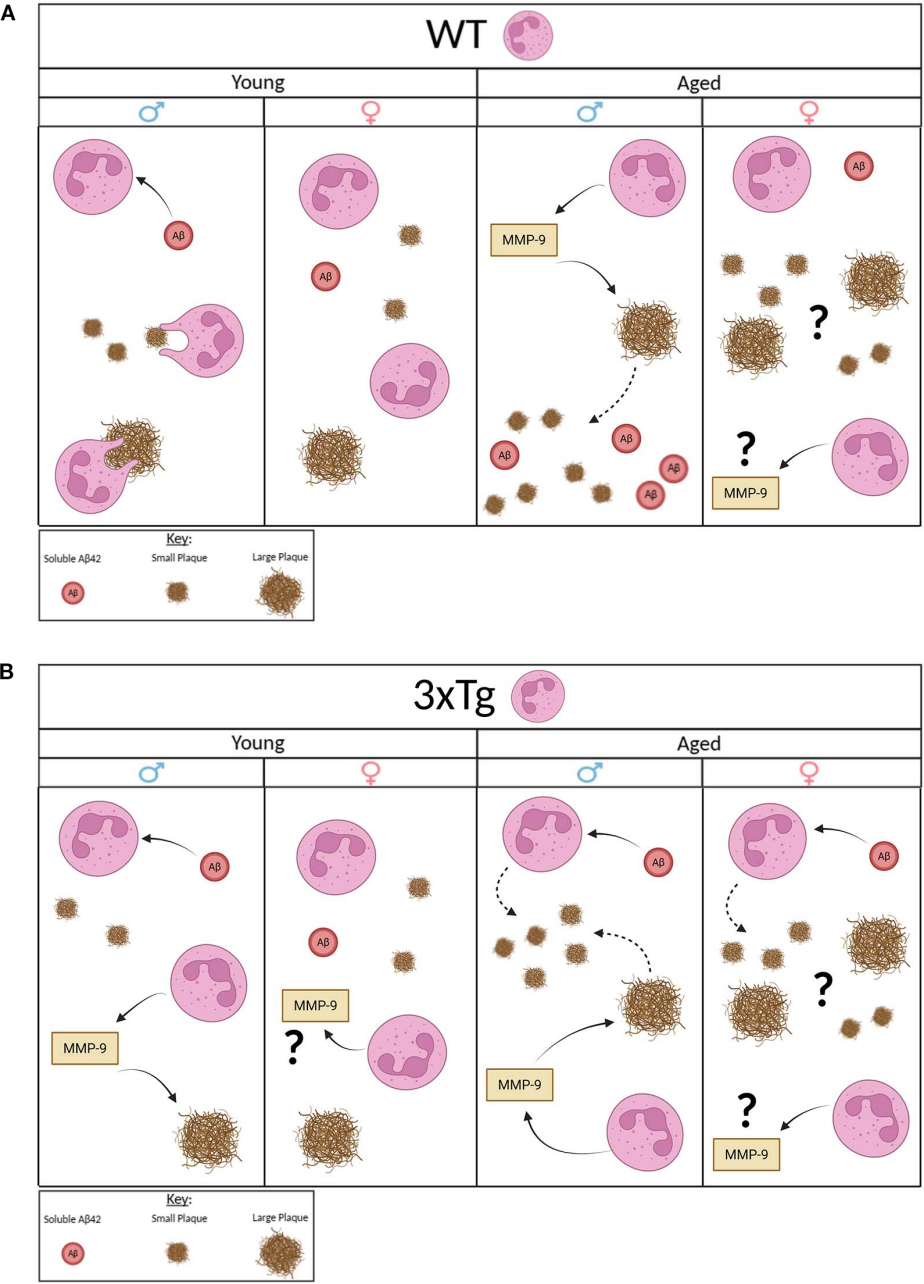
Proposed model. Young WT male neutrophils clear soluble Aβ and reduce the quantity of small and large plaques. Young WT female neutrophils have no effect on Aβ. Aged WT male mice highly express MMP-9 that potentially degrades large plaques and increases presence of small plaques and soluble Aβ. Aged WT female mice release high MMP-9 but increase presence of small and large plaques with no effect on soluble Aβ (A.). Young 3xTg male neutrophils clear soluble Aβ and release high MMP-9 that potentially degrades large plaques with no effect on small plaques. Young 3xTg female neutrophils release high MMP-9 with no effect on Aβ. Aged 3xTg male neutrophils take up soluble Aβ and release high MMP-9 that potentially degrades large plaques but increases the presence of small plaques. Aged 3xTg female neutrophils take up soluble Aβ and release high MMP-9 but with an increase in the quantity of small and large plaques. Image generated using BioRender.com.

Our data show a clear sex and genotype difference in neutrophils interaction with Aβ. Our data from WT mice show that male and female derived neutrophils have opposing effects on Aβ levels *in vitro*. Our data suggest that neutrophils derived from young male mice are efficient at reducing soluble Aβ and insoluble plaques of all sizes (**Figure 5A**). Those from aged male mice efficiently degrade large plaques which leads to the production of more soluble Aβ and an increase in small plaques. A process that is potentially mediated by the release of MMP-9 in aged male neutrophils. MMP-9 was previously shown to degrade Aβ peptides (21). On the other hand, female derived neutrophils, young and aged, show a clear deficit in modulating Aβ levels (**Figure 5A**). Interestingly, neutrophils derived from aged female mice show an increased release of MMP-9 and an increase in both small and large plaques in culture. We postulate that aged female neutrophils may release factors that stimulate the increased production of Aβ by neurons. We propose that these sex differences in function may contribute to the sex bias observed in sporadic AD, a large AD subset with no clear genetic underpinning.

As for neutrophils derived from 3xTg mice, an AD model, sex and age differences are observed in Aβ modulation (**Figure 5B**). Our data show that neutrophils from young 3xTg male mice internalize soluble Aβ and decrease large plaques through the release of MMP-9. Interestingly, no change was observed in the density of small plaques. Neutrophils from aged male mice are efficient at removing soluble Aβ and breaking down large plaques, as observed in the presence of young 3xTg neutrophils. However, aged male neutrophils led to an increase in the number of small plaques in culture. This suggest that these cells are unable to degrade internalized Aβ. This failure leads to the generation of small extracellular plaques by these cells. A process previous shown to be performed by microglia (22). As for neutrophils from female young 3xTg mice, they have no effect on soluble Aβ or plaques even with an increase in MMP-9 release. Interestingly, neutrophils from aged female 3xTg mice can reduce soluble Aβ levels. The increase in both small and large plaques would suggest that these neutrophils also generate extracellular plaques after internalization of soluble fragment. Overall, these data, from both WT and 3xTg mice suggest a functional deficit in phagocytosis by neutrophils with age and disease state, a phenotype observed in neutrophils isolated from AD patients (8, 9). Additionally, it is well established that soluble Aβ fragments are more toxic than aggregates (23, 24). Therefore, our data showing aged neutrophils modulation of soluble Aβ (WT aged male neutrophils increase levels; aged 3xTg male and female reduce levels) could indicate a potential role these cells play in disease pathology.

Neutrophils from aged AD patients (average age 67.4 years) and APP/PS1 mice (aged 12 months) show increased ROS production and NETosis (19) suggesting that neutrophils contributed to the inflammation observed in the AD brain. Though our data did not show an increase in cytokine production in neutrophils from aged 3xTg mice, a clear increase was observed in free DNA present in culture. Our data from young mice suggest a high inflammatory state of these cells. It is interesting that this inflammatory profile diminished completely with age, whether this change affects disease progression is unknown. Finally, Although MPO was observed in plaques in human AD brains, neutrophil extracellular traps were only localized to the brain vasculature (19). Our data shows that in the presence of AD neurons, neutrophils from 3xTg mice release NETs. Since neutrophils from WT mice do not release NETs in the presence of 3xTg neurons, we postulate that the increase in NETosis could be an indication of further dysregulation of neutrophil function in 3xTg mice. Many reports of neutrophils in various AD model characterize these cells in the aged brain microvasculature (3, 19). Therefore, an importance of the work presented here is that our data show that neutrophils from young AD mice, before symptoms occur, already show functional dysregulation. While we cannot conclusively say inflammatory state observed the co-culture system is from neutrophils directly, our data provide evidence that neutrophils are important mediators of the increased inflammation. This is also supported by the increased release of neutrophils from the bone marrow of young 3xTg mice.

Our data showed a change in the inflammatory profile of neuronal cultures after neutrophil addition. Aβ has been shown to act as a proinflammatory cytokine by signaling through TLR4 and RAGE receptors (25). Because these neuronal cultures release Aβ, it is possible that the inflammatory changes observed is a result of Aβ signaling through these receptors. It is also possible that the isolated neutrophils are inherently abnormal due to the disease state. These possibilities will need further investigation.

This work has several limitations. Our *in vitro* system is a ratio of 11 neutrophils for every neuron. The ratio of neutrophils to neurons in the AD brain is unknown, therefore it is unclear how to translate our results to these models. Neutrophils were isolated from the bone marrow for this study. The function of bone marrow neutrophils does not necessarily correlate to the function of neutrophils in the blood or AD brain. We recognize that the migration of neutrophils into the blood and the brain changes neutrophil physiology. We stipulate that if changes are observed in the more naïve state, bone marrow neutrophils, then changes will be present in their function in the blood and the brain. In addition, we chose to use bone marrow neutrophils to minimize the number of mice used to perform the experiments presented here. This study does not address tau hyperphosphorylation, a critical pathology of AD and a product of a mutation in 3xTg mice. tau hyperphosphorylation is observed late in the 3xTg mouse, after 12 months of age. Interestingly, staining of our primary neuronal cultures showed no tau hyperphosphorylation. This would suggest that these neurons were not senescent enough to show this phenotype. It is therefore possible that aging these cultures past 4 weeks may provide a model to study neutrophil physiology in the presence of both Aβ and tau hyperphosphorylation.

## Conclusion

This study showed that neutrophils from AD mice are functionally different than neutrophils from WT mice. Our data showed a sex difference in how neutrophils interact with Aβ in the presence of neurons. The observed sex difference is age dependent (only present in neutrophils from young mice). Whether this contributes to the sex bias observed in AD (both mice and humans) remains to be determined. Our results showed that neutrophils from young and age 3xTg mice have altered interact with debris and release of immune modulators. We postulate that neutrophil dysfunction underlies the detrimental contributions of neutrophils to AD pathology.

## Data Availability

The original contributions presented in the study are included in the article/**Supplementary Material**. Further inquiries can be directed to the corresponding author.

## Supplementary material

Supplementary Figure 1 Validation of neutrophil isolation and 3xtg neuronal cultures. The neutrophil isolation protocol enriched neutrophil purity to 80%. The remaining 20% of cells consisted of blasts, Ly6gG- granulocytes, lymphocytes, and monocytes **(A)**. Both beta-tubulin+ and GFAP+ cells were detected in primary 3xTg cultures (**B**, scale bar=50µm). GFAP+ cells were on average 15% of cells in culture **(C)**. More neutrophils were isolated from the bone marrow of young WT mice compared to young 3xTg mice and aged mice of both genotypes **(D)** Flow cytometry showed that young 3xTg mice had more Ly6G+ cells in their blood **(E)**. Representative characterization, using IMARIS, of Abeta inside (<0µm away) or outside (>1µm away) neutrophils (Ly6B+) (**F**, scale bar=10µm). 80% of Aβ was observed to be external of beta-3-tubulin staining **(G)**. Aged neutrophils showed a decrease in the percentage of neutrophils with internalized Aβ in 3xTg primary neuronal cultures **(H)** In bar graphs, red dots are cultures exposed to female-derived cells and blue dots are those exposed to male-derived cells. Comparisons performed using Welch’s unpaired T-test or one-way ANOVA. Two-way ANOVA was performed followed by Fisher’s least significant difference test for post-hoc analysis **(H)** *, p<0.05.

Supplementary Figure 2 Neutrophil have little effect on levels of soluble Aβ-40 in 3xTg primary neuronal cultures. 3xTg primary neuronal cultures showed little change in soluble Aβ40 levels **(A)**. The addition of neutrophils to the cultures had no effect on Aβ-40 levels **(B)**. Only neutrophils derived from young male 3xTg mice increased the levels of Aβ-40 in culture **(C)**. Neutrophils from female mice had no effect on Aβ-40 levels **(D)**. Each dot represents data from pup culture exposed to neutrophils isolated from either male or female, WT or 3xTg mice. One-way ANOVA was performed followed by Dunnett’s multiple comparison test was performed for post-hoc analysis. In bar graphs, red dots are cultures exposed to female-derived cells and blue dots are those exposed to male-derived cells. *, p<0.05.

Supplementary Figure 3 Aging decreases the number of neutrophils phagocytosing Aβ in vitro. Quantity of small plaques (<200 voxels) was decreased by neutrophils from young WT male mice, with no effect from neutrophils from young 3xTg male mice. the number of large fragments (<200 voxels) was decreased by neutrophils from young WT and 3xTg mice **(A)**. Neutrophils from young WT and 3xTg female mice had no effect on the number of small or large plaques in culture **(B)**. The number of small plaques were increased in the presence of neutrophils from aged WT and 3xTg male mice. Only neutrophils from aged WT male mice decreased the number of large plaques in culture **(C)**. The presence of aged WT and 3xTg female neutrophils increased the number of small and large plaques in culture **(D)**. Figures **(C–F)** show average for the data presented in figures 2 **(C, E)** and 3 **(B, D)**.

Supplementary Figure 4 Neutrophil from CVN mice, another AD rodent model, have impaired removal of cellular debris. Neutrophils isolated from young CVN mice and incubated in culture plates with neuronal debris (**A**, red arrow points to large debris and white arrow points to small debris) showed that neutrophils derived from male and female CVN mice fail to remove cellular debris when compared to their WT counterparts **(B)**. Data is representative of 4 pup cultures exposed to neutrophils from WT male and female mice, and 6 pup cultures exposed to neutrophils from CVN male and female mice. scale bar=50µm. Two-way ANOVA was performed followed by Dunnett’s multiple comparisons test.

Supplementary Figure 5 The presence of neutrophils had little effect on the levels of growth factors and chemokines in 3xTg primary neuronal cultures. The addition of neutrophils had little effect on neuronal density in culture **(A)**. The addition of neutrophils from young 3xTg male mice increased the release of G-CSF in culture. Young male and female 3xTg neutrophils decreased the release of CXCL1 in culture while aged 3xTg females decreased the release of CXCL12 **(B)**. Each dot represents data from a pup culture exposed to neutrophils isolated from either male or female, WT or 3xTg mice. One-way ANOVA was performed followed by Tukey’s multiple comparison test for post-hoc analysis. *, p<0.05.
