# Supplementary material for: Neutrophils from Alzheimer’s disease mice fail to phagocytose debris and show altered release of immune modulators with age
Source: Front Immunol. 2025 Oct 20;16:1672768. doi: 10.3389/fimmu.2025.1672768 (PMC12580139; doi:10.3389/fimmu.2025.1672768)
Supplement: Supplementary Figure 1 [file Presentation1.pdf]

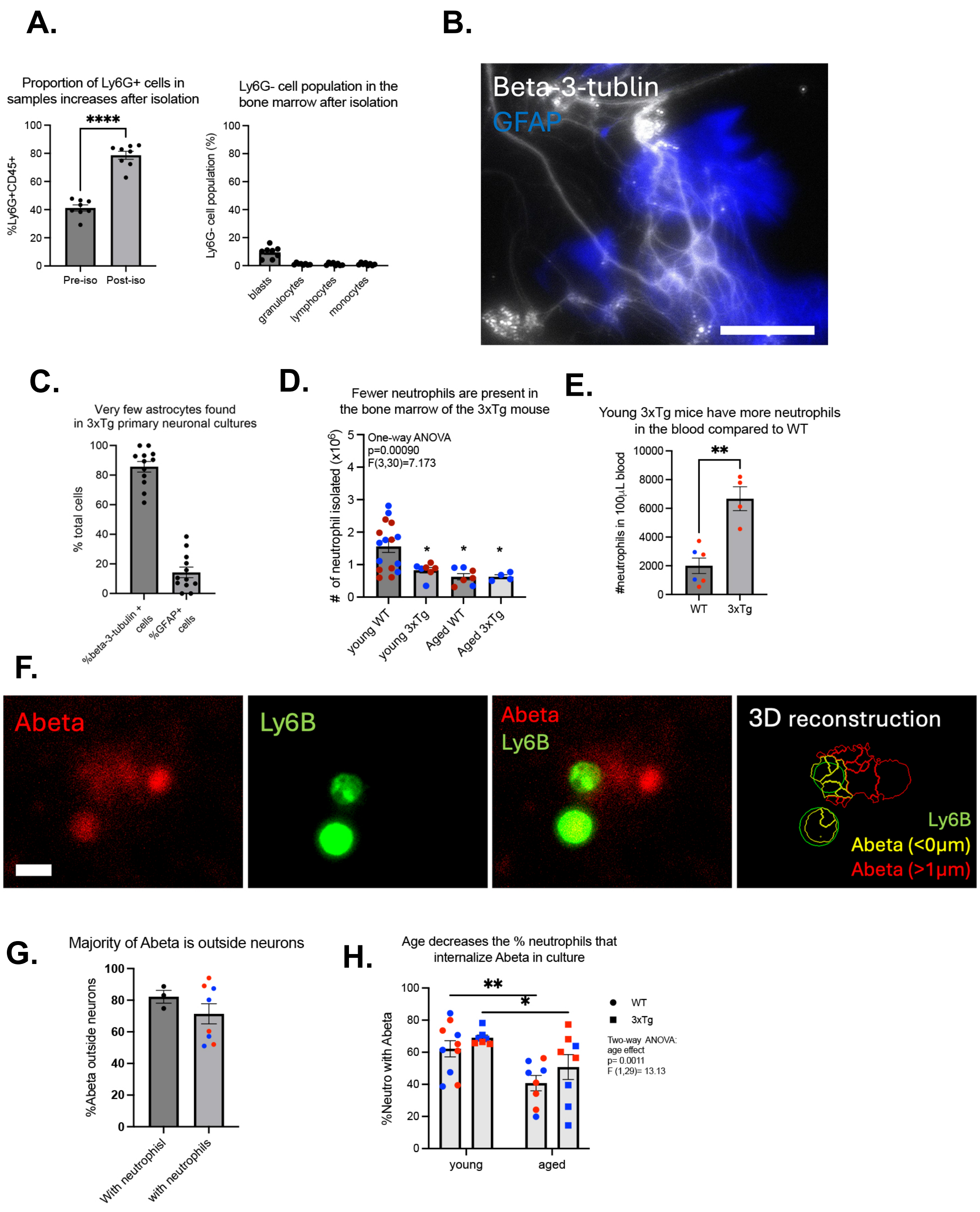

Supplemental figure 1

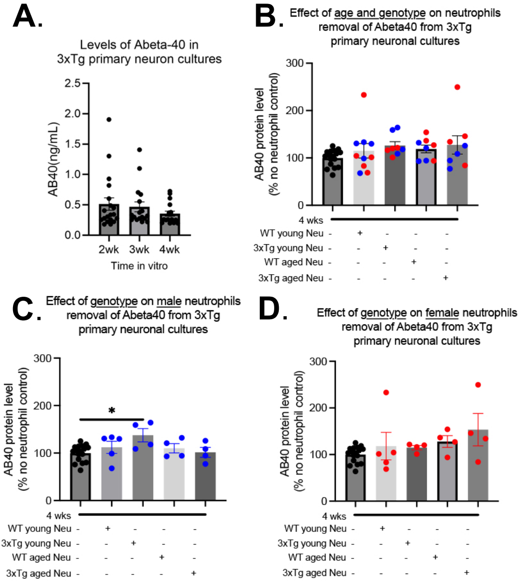

Supplemental Figure 2

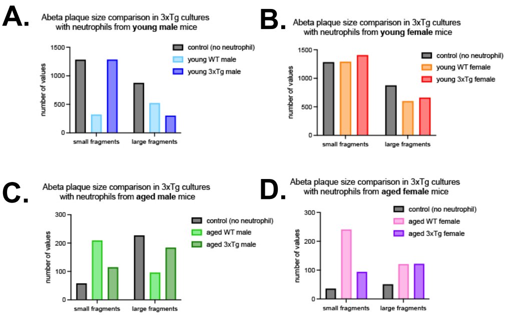

Supplemental Figure 3

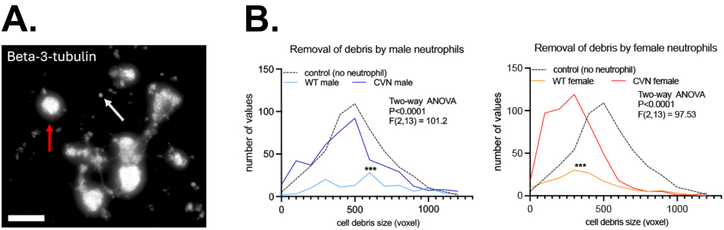

Supplemental figure 4

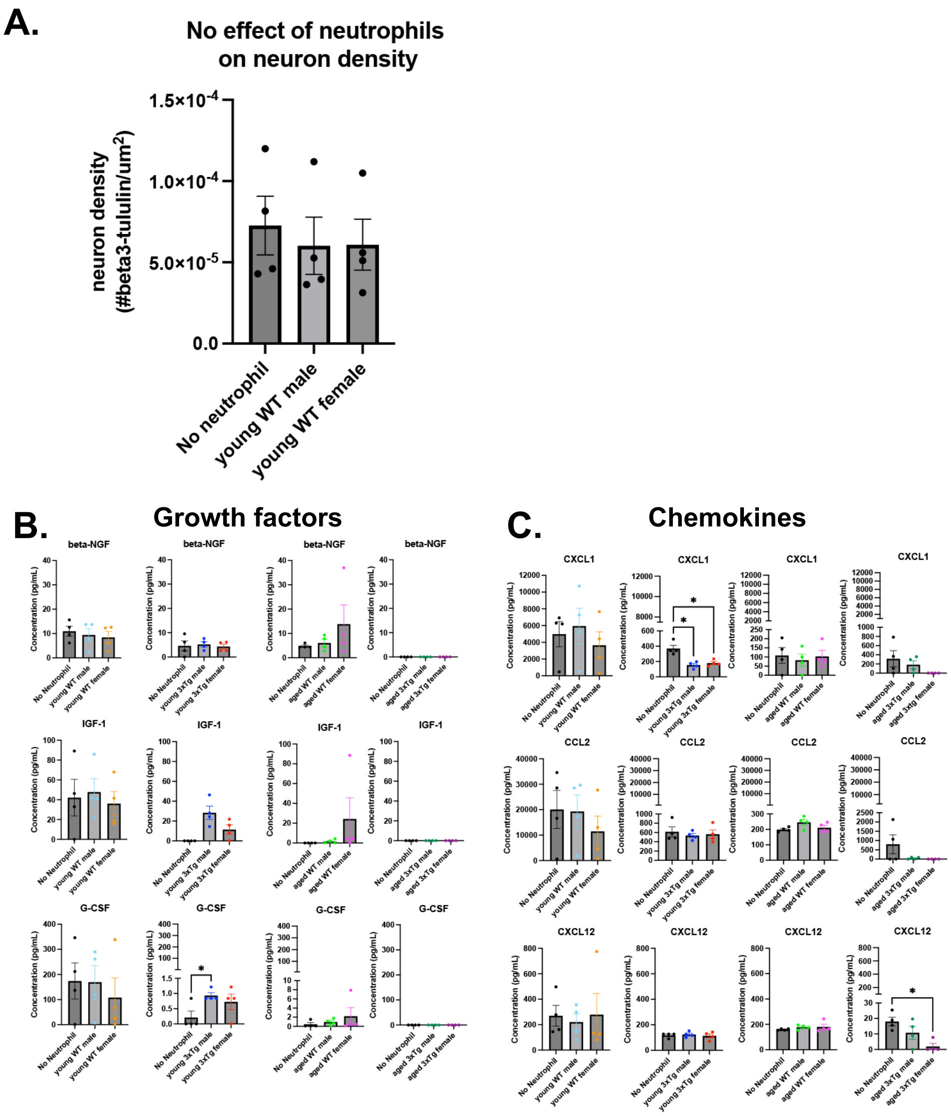

Supplemental figure 5

<u>Supplemental figure 1:</u> Validation of neutrophil isolation and 3xtg neuronal cultures. The neutrophil isolation protocol enriched neutrophil purity to 80%. The remaining 20% of cells consisted of blasts, Ly6gG- granulocytes, lymphocytes, and monocytes (A.). Both beta-tubulin+ and GFAP+ cells were detected in primary 3xTg cultures (B., scale bar=50µm). GFAP+ cells were on average 15% of cells in culture (C.). More neutrophils were isolated from the bone marrow of young WT mice compared to young 3xTg mice and aged mice of both genotypes (D.) Flow cytometry showed that young 3xTg mice had more Ly6G+ cells in their blood (E.). Representative characterization, using IMARIS, of Abeta inside (<0µm away) or outside (>1µm away) neutrophils (Ly6B+) (F., scale bar=10µm). 80% of Aβ was observed to be external of beta-3-tubulin staining (G.). Aged neutrophils showed a decrease in the percentage of neutrophils with internalized Aβ in 3xTg primary neuronal cultures (H.). In bar graphs, red dots are cultures exposed to female-derived cells and blue dots are those exposed to male-derived cells. Comparisons performed using Welch's unpaired T-test or one-way ANOVA. Two-way ANOVA was performed followed by Fisher's least significant difference test for post-hoc analysis (H.) *, $p<0.05$.

<u>Supplemental figure 2</u>: Neutrophil have little effect on levels of soluble Aβ-40 in 3xTg primary neuronal cultures. 3xTg primary neuronal cultures showed little change in soluble Aβ40 levels (A.). The addition of neutrophils to the cultures had no effect on Aβ-40 levels (B.). Only neutrophils derived from young male 3xTg mice increased the levels of Aβ-40 in culture (C.). Neutrophils from female mice had no effect on Aβ-40 levels (D.). Each dot represents data from pup culture exposed to neutrophils isolated from either male or female, WT or 3xTg mice. One-way ANOVA was performed followed by Dunnett's multiple comparison test was performed for post-hoc analysis. In bar graphs, red dots are cultures exposed to female-derived cells and blue dots are those exposed to male-derived cells. *, $p<0.05$.

<u>Supplemental figure 3:</u> Aging decreases the number of neutrophils phagocytosing Aβ in vitro. Quantity of small plaques (<200 voxels) was decreased by neutrophils from young WT male mice, with no effect from neutrophils from young 3xTg male mice. the number of large fragments (>200 voxels) was decreased by neutrophils from young WT and 3xTg mice (A.). Neutrophils from young WT and 3xTg female mice had no effect on the number of small or large plaques in culture (B.). The number of small plaques were increased in the presence of neutrophils from aged WT and 3xTg male mice. Only neutrophils from aged WT male mice decreased the number of large plaques in culture (C.). The presence of aged WT and 3xTg female neutrophils increased the number of small and large plaques in culture (D.). Figures (C-F) show average for the data presented in figures 2 (C and E) and 3 (B and D).

<u>Supplemental figure 4:</u> Neutrophil from CVN mice, another AD rodent model, have impaired removal of cellular debris. Neutrophils isolated from young CVN mice and incubated in culture plates with neuronal debris (A., red arrow points to large debris and white arrow points to small debris) showed that neutrophils derived from male and female CVN mice fail to remove cellular debris when compared to their WT counterparts (B.). Data is representative of 4 pup cultures exposed to neutrophils from WT male and female mice, and 6 pup cultures exposed to neutrophils from CVN male and female mice. scale bar=50µm. Two-way ANOVA was performed followed by Dunnett's multiple comparisons test.

<u>Supplemental figure 5:</u> The presence of neutrophils had little effect on the levels of growth factors and chemokines in 3xTg primary neuronal cultures. The addition of neutrophils had little effect on neuronal density in culture (A.). The addition of neutrophils from

young 3xTg male mice increased the release of G-CSF in culture. Young male and female 3xTg neutrophils decreased the release of CXCL1 in culture while aged 3xTg females decreased the release of CXCL12 (B.). Each dot represents data from a pup culture exposed to neutrophils isolated from either male or female, WT or 3xTg mice. One-way ANOVA was performed followed by Tukey's multiple comparison test for post-hoc analysis. *, $p<0.05$.
